# Cox Regression Based Modeling of Functional Connectivity and Treatment Outcome for Relapse Prediction and Disease Subtyping in Substance Use Disorder

**DOI:** 10.3389/fnins.2021.768602

**Published:** 2021-11-11

**Authors:** Tianye Zhai, Hong Gu, Yihong Yang

**Affiliations:** Neuroimaging Research Branch, Intramural Research Program, National Institute on Drug Abuse, National Institutes of Health, Baltimore, MD, United States

**Keywords:** prediction modeling, fMRI, treatment outcome, Cox regression, functional connectivity, neuromodulation implications

## Abstract

Functional magnetic resonance imaging (fMRI) has become one of the most widely used noninvasive neuroimaging technique in research of cognitive neurosciences and of neural mechanisms of neuropsychiatric/neurological diseases. A primary goal of fMRI-based neuroimaging studies is to identify biomarkers for brain-behavior relationship and ultimately perform individualized treatment outcome prognosis. However, the concern of inadequate validation and the nature of small sample sizes are associated with fMRI-based neuroimaging studies, both of which hinder the translation from scientific findings to clinical practice. Therefore, the current paper presents a modeling approach to predict time-dependent prognosis with fMRI-based brain metrics and follow-up data. This prediction modeling is a combination of seed-based functional connectivity and voxel-wise Cox regression analysis with built-in nested cross-validation, which has been demonstrated to be able to provide robust and unbiased model performance estimates. Demonstrated with a cohort of treatment-seeking cocaine users from psychosocial treatment programs with 6-month follow-up, our proposed modeling method is capable of identifying brain regions and related functional circuits that are predictive of certain follow-up behavior, which could provide mechanistic understanding of neuropsychiatric/neurological disease and clearly shows neuromodulation implications and can be used for individualized prognosis and treatment protocol design.

## Introduction

Since the initial demonstration of blood-oxygen-level-dependent (BOLD) signal *in vivo* in early 1990s (Ogawa et al., 1990), functional magnetic resonance imaging (fMRI) has become a noninvasive neuroimaging technique widely used in research of cognitive neurosciences, as well as in understanding of neural mechanisms of neuropsychiatric/neurological diseases. For example, in exploring neurobiological mechanisms of substance use disorder, a highly relapsing chronic brain disease (Dutra et al., 2008; Koob and Volkow, 2016; Volkow et al., 2016) currently without effective treatments, imaging biomarkers based on both resting-state fMRI (Zhai et al., 2021) and task-evoked fMRI (Luo et al., 2013) have shown prediction validity of relapse to cocaine use following treatment. Such neuroimaging-based studies provide mechanistic understanding of relapse to drug use and suggest neural targets for the development of neuromodulation (e.g., transcranial magnetic stimulation/TMS) treatment protocols.

Over the past three decades, in spite of advances in both imaging acquisition and analysis techniques, which have greatly enhanced our understanding of brain function and dysfunction, two major concerns have been associated with fMRI-based neuroimaging studies that hinder the translation from scientific knowledge to clinical practice. One concern is lack of appropriate validation in fMRI data analyses. A primary goal of fMRI-based neuroimaging research is to identify biomarkers that can be used to establish the relationship between brain and behavior and ultimately perform individualized predictions of health/prognosis outcomes (Gabrieli et al., 2015; Shen et al., 2017). However, the term of “prediction” was often misused when actual results reported were association/correlation that related brain measures with behavioral assessments/treatment outcome within samples being tested (Meng et al., 2017; Shen et al., 2017). A recent review on neuroimaging studies claiming prediction validity using fMRI data indicates that among 100 studies from 2017 to 2019, over 40% claimed prediction without any cross-validation (Poldrack et al., 2020). These in-sample correlational models without proper cross-validation tend to provide inflated prediction accuracy due to overfitting, resulting in difficulties in generalizability (Whelan and Garavan, 2014; Shen et al., 2017). Another concern is the relatively small sample size in fMRI-based neuroimaging studies (Turner et al., 2018; Szucs and Ioannidis, 2020), partially due to the large financial expenses associated with fMRI scanning, as well as difficulties associated with recruiting certain types of patients/participants. Among the 100 studies examined, more than 70% were with a sample size less than 100, and more than 50% were with a sample size less than 50 (Poldrack et al., 2020). The small sample size nature of fMRI-based neuroimaging studies can further intensify the problem of overfitting in prediction analyses (Whelan and Garavan, 2014). Previous studies have suggested the utilization of nested cross-validation in prediction, which has been demonstrated to be able to provide robust and unbiased model performance estimates, and outperform some other commonly used cross-validation methods (such as K-fold cross-validation) especially in applications with small sample sizes (Varma and Simon, 2006; Vabalas et al., 2019).

Therefore, we present here an analytical approach to predict time-dependent follow-up behaviors by imaging metrics from resting-state fMRI and demonstrated the approach in a cohort of treatment-seeking cocaine users. Our prediction modeling is a hybrid of hypothesis-driven and data-driven approaches built upon a combination of seed-based functional connectivity and voxel-wise Cox regression. The seed-based functional connectivity serves as the hypothesis-driven part, which is hypothesis specific (e.g., implication for neuromodulation target selection) and ensures the interpretability of results. The Cox regression was originally introduced for survival analysis (Cox, 1972). Due to the mathematical similarity between survival and the relapse to drug use (time-dependent binary outcome), the Cox regression model is an ideal statistical tool for probing brain—relapse relationship. For example, it has been utilized in predicting cocaine relapse with the brain activation induced by a stop-signal inhibition task as measured by fMRI (Luo et al., 2013). Also utilized under the resting-state, the Cox model yielded high accuracy in predicting cocaine relapse with functional connectivity (Geng et al., 2017). Therefore, we choose the Cox regression model for the study of relapse during follow-up after treatment. The whole prediction modeling pipeline is organized into a nested cross-validation loop. Detailed procedures are described below.

## Materials and Equipment

The prediction method that we proposed here is based on a voxel-wise Cox regression of resting-state fMRI and treatment outcome. The whole procedure is cross-validated, potentially utilizable on novel patients/subjects to predict their treatment outcome prospectively. Here we list all the materials and equipment that we used to conduct the prediction modeling: A functional magnetic resonance imaging (fMRI) dataset acquired from an MRI scanner and a post-treatment follow-up dataset of relapse to drug use. Tools for image pre-processing and functional connectivity analyses include the AFNI (v17.0.06^1^) and SPM^2^ software packages. The computational pipeline of relapse prediction, including voxel-wise Cox regression, prediction model fitting, cross-validation, and *post-hoc* analyses, were developed with Matlab (R2020b, The MathWorks, Inc., Natick, MA, United States).

### Methods

#### Participants and Clinical Assessment Procedures

To demonstrate our relapse prediction modeling, we employed imaging and behavioral data collected from a cohort of 45 treatment-seeking cocaine dependent participants who underwent and completed psychosocial treatment from local residential treatment programs using the Minnesota Model Psychosocial treatment approach (Cook, 1988). Several clinical measurements were assessed including the Inventory of Drug Use Consequences (InDUC) which assess the life problems related to drug use (Tonigan and Miller, 2002), Cocaine Craving Questionnaire (CCQ-Brief), years of cocaine use, days of cocaine use in the past 90 days, and days since last cocaine use. Following discharge from the psychosocial treatment program, participants were followed up for 168 days or until relapse, whichever was earlier. Abstinence was verified by weekly phone interviews and/or in-person urine drug screens. Date of relapse was recorded as the day of drug use or the day of the first missed appointment if lost to follow-up. Participants who failed to maintain abstinence were then discharged from the study. The study was reviewed and approved by the Institutional Review Boards of the University of Texas Southwestern Medical Center and the Veterans Administration North Texas Health Care System. Written informed consent was obtained from each participant. Summary of the demographic information of the cohort is presented in Table 1 (*n* = 43 after excluding two participants for excessive head motion during fMRI scanning, see section “Computational Pipeline of Relapse Prediction” in the section “Methods”). More detailed information on participants’ inclusion/exclusion, treatment/assessment procedures have been described previously (Zhai et al., 2021).

**TABLE 1:** Demographic, clinical and head-motion assessments.

	**CD cohort (*n* = 43)**
Age	43.42 ± 7.19
Sex (M/F)	38/5
Edu (years)	12.49 ± 2.11
Cocaine use (years)	8.28 ± 5.22
Nicotine use (CPD)	11.42 ± 10.36
Mean head motion (mm)	0.09 ± 0.03

*CD, cocaine dependent; Edu, years of education; CPD, cigarettes per day.*

#### Magnetic Resonance Imaging/Functional Magnetic Resonance Imaging Scanning Parameters

For each participant, a whole-brain BOLD resting-state fMRI dataset of 6 min was acquired from a 3T Philips MRI scanner with an eight-channel radio-frequency coil (Philips Medical Systems, Best, Netherlands). Images were collected in the axial plane parallel to the AC-PC line using a single-shot, echo-planar imaging sequence (TE = 25 ms, TR = 1.7 s, flip angle = 70°, spatial resolution = 3.25 mm × 3.25 mm × 3 mm with no gap). Participants were instructed to keep their heads still and eyes open during the resting-state scan. A high-resolution anatomical T1-weighted image was also acquired from each participant using a 3D magnetization-prepared rapid gradient-echo sequence (TE = 3.8 ms, TR = 8.2 ms, flip angle = 12°, spatial resolution = 1 mm × 1 mm × 1 mm).

#### Computational Pipeline of Relapse Prediction

**FIGURE 1:**
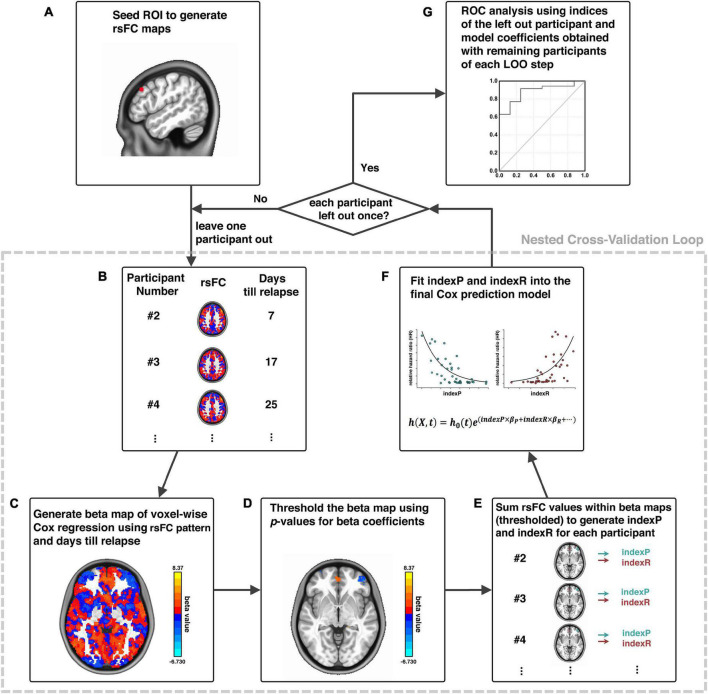
Schematic diagram of analytical pipeline. Illustration of our analytical procedures using one exemplar seed ROI, adapted from Zhai et al. (2021). First, an ROI is selected as a seed **(A)**; the whole-brain rsFC of this seed is calculated for each participant **(B)**; a voxel-wise Cox regression is conducted using rsFC and days until relapse to generate beta maps **(C)**; beta maps of Cox regression is thresholded **(D)**; generation of indexP and indexR by linearly summation of the rsFC values within the thresholded beta maps for the negative and positive beta voxels, respectively **(E)**; construction of the final prediction model by fitting indexP and indexR into the Cox model **(F)**; procedures B to F were organized in a nested cross-validation loop, and after each participant is left out once, an ROC analysis evaluates the final prediction model **(G)**. rsFC, resting-state functional connectivity; ROI, region-of-interest; ROC, Receiver-Operating-Characteristics.

The relapse prediction modeling that we developed and demonstrated here was inspired by the connectome-based predictive modeling proposed by Shen et al. (2017). Generally, our methodological pipeline was a combination of seed-based functional connectivity and voxel-wise Cox regression of functional connectivity and treatment outcome in a nested cross-validation fashion, which consisted of six logical elements: (1) image preprocessing; (2) functional connectivity calculation; (3) voxel-wise Cox regression analysis; (4) thresholding and generating composite indices; (5) Cox model fitting for brain-behavior relationship and model evaluation (ROC analysis); and (6) cross validation and permutation test. Procedural steps are illustrated in Figure 1 and described in details below.

The first step was image pre-processing that included: discarding the first five volumes to allow the magnetic resonance signal to reach steady state, slice timing correction (*3dTshift, AFNI*), volume registration (*3dvolreg, AFNI*), polynomial detrending (up to the 3rd order, *3dDetrend, AFNI*) and head motion correction (*3dTproject, AFNI*). Signals from white matter and cerebrospinal fluid (CSF) were treated as a marker of non-neuronal noise and were regressed out (*3dTproject, AFNI*). A band-pass filter was applied to select low-frequency fluctuations between 0.012 and 0.1 Hz (*3dTproject, AFNI*) (Fransson, 2005). The fMRI data were normalized to standard MNI image space and resampled to a 2-mm isotropic resolution (SPM12). Head motion was also evaluated at the frame-by-frame level to further control for image quality using pair-wise displacement calculated based on the Euclidean distance (*1d_tool.py, AFNI*). Volumes with displacement >0.35 mm were censored; participants were excluded if their mean head motion across volumes were greater than 0.2 mm or their percentage of censored volumes exceeding 20%. Two participants were excluded due to head motion exceeding this threshold, leaving 43 participants in the subsequent analytical steps.

Step two was to select a seed or region-of-interest (ROI) based on specific hypothesis and calculate its whole-brain functional connectivity (Figure 1A). The dlPFC has been utilized as stimulation target for high frequency rTMS treatment that reduced craving for nicotine and cocaine (Politi et al., 2008; Li et al., 2013; Pripfl et al., 2014). Here, we chose an ROI on the left dlPFC for demonstration due to its promising role in the treatment of cocaine dependence as stimulation target of high frequency rTMS (Terraneo et al., 2016), as well as the high validity in predicting cocaine relapse with its downstream functional circuits (Zhai et al., 2021). Whole-brain functional connectivity maps were obtained by calculating the cross-correlation (CC) between the time series of the seed and that of each voxel in the whole-brain (*3dDeconvolve, AFNI*). The CC maps were then Fisher’s Z-transformed with the inverse hyperbolic tangent function *Z* = *atanh*(*cc*) (*3dcalc, AFNI*) as the resting-state functional connectivity (rsFC) maps for subsequent analyses.

Step three was to conduct the voxel-wise Cox regression analysis on the rsFC generated in the previous step and the relapse information (days till relapse) obtained during the follow-up period (Figure 1B): *h*(*X*_*i*_,*t*) = *h*_0_(*t*)*e*^∑*_j_x*_*ij*_*b*_*j*_^, where *X_i_* is the linear combination of the predictor variables/covariates for the *i*th participant *h*(*X*_*i*_,*t*), is the hazard rate at time *t* for *X_i_*, *h*_0_(*t*) is the baseline hazard rate function, and *x*_*ij*_*b*_*i*_ within the exponential term represents the loglinear regression. The voxel-wise beta coefficient maps (beta map) of all participants were obtained in this step (Figure 1C), and the whole-brain relative hazard ratio (HR) maps can further be calculated with the exponential of the beta values.

Step four was to generate composite indices. All beta/HR maps were voxel-wisely thresholded at a given threshold (e.g., *p* < 0.001 as demonstrated here). This is an initial thresholding serving the purpose of pre-selection of those voxels with functional connectivity relates to relapse the most, and was arbitrarily chosen based on empirical experience, similarly as demonstrated in the connectome-based predictive modeling (Shen et al., 2017). These thresholded beta/HR maps were subsequently used to generate a set of “protective circuits” (voxels with negative beta values/HR value less than 1, indicating less risk of relapse with stronger functional connectivity), and a set of “risk circuits” (voxels with positive beta values/HR value greater than 1, indicating higher risk of relapse with stronger functional connectivity), as well as two composite indices: indexP and indexR by linear summation of functional connectivity from all voxels within the “protective” and the “risk” circuits, respectively (Figures 1D,E).

Step five was the final Cox model fitting for brain-behavior relationship using these two composite indices, with age, sex, years of education (edu), daily cigarette use (CPD) and head motion during fMRI scan (HM) as covariates: *h*(*X*_*i*_,*t*) = *h*_0_(*t*)e^(*indexP*×β_*P*_+*indexR*×β_*R*_ + *age*×β_*A*_+*sex*×β_*S*_^^+^^*edu*×β_*E*_+*CPD*+β_*C*_+*HM*×β_*H*_)^, (Figure 1F), and Receiver-Operating-Characteristic (ROC) analysis for model evaluation (Figure 1G). Step three to step five (Figures 1B–F) was organized into a nested cross-validation loop, where the voxel-wise Cox regression and thresholding serve the purpose of feature selection, and the final Cox fitting with the two composite indices works as final model generation. The loop was repeated *n* times (*n* equals the number of participants), and each time a new model was developed from scratch with the *n-*th participant being left out. After the nested cross-validation loop was completed, the thresholds array for the cross-validated ROC analysis for model evaluation was generated with the actual predictor values (i.e., indexR and indexP, as well as other covariates such as age and years of education etc.) of each participant and the model that was generated with this participant being left out (Figure 1G).

In the final step, permutation test was performed to determine statistical significance based on empirical distribution determined with the permutation. We repeated the entire analysis 10,000 times, each time with the predictor (composite indices) and outcome (days till relapse) pairs randomly permuted to generate the data/model specific empirical *null* distribution for the area-under-the-curve (AUC) values of the ROC curve. The *p*-value of the AUC was then derived based on the ranking of the actual AUC value in this empirical *null* distribution.

Furthermore, our prediction modeling can be adapted into different settings based on specific applications. For example, in order to evaluate the prediction potential of our composite indices in early relapse prediction, we generated an early relapse prediction model with the indexP and indexR to predict cocaine relapse at an early follow-up cut-off of 30-day follow-up, as well as an intermediate prediction model with follow-up cut-off at 90-day, using the pipeline described above.

#### *Post-hoc* Analyses of Disease Subtyping

We performed several *post-hoc* analyses to further utilize our prediction modeling to explore brain mechanisms of cocaine addiction and to assess individual difference in the brain (functional connectivity) versus behavioral (relapse) relationship. First, we binarized the HR maps of all leave-one-out steps, and then stacked them together to generate a heat map of relapse relevant functional circuits. This heat map was further arbitrarily thresholded based on the majority of leave-one-out steps (e.g., > 85% as demonstrated here) to demonstrate the group-level protective and risk circuits. We then extracted the averaged functional connectivity from each of the 43 participants within the group-level protective and risk circuits as the input for our *post-hoc* analyses, defined as the idxP_ph, and the idxR_ph, respectively, for the protective and risk circuits (note here the suffix “ph” stands for *post-hoc*, to be differentiated from the composite indices “indexP” and “indexR” used in the abovementioned prediction modeling section). Linear regression analysis was conducted to explore the relationship between the idxP_ph and the idxR_ph. To identify potential subtypes of cocaine dependence, we also conducted the *k*-means clustering in the P-R space (idxP_ph and idxR_ph). The optimal number of clusters was determined by visual inspection with the “elbow criterion” at a cluster number so that adding another cluster only grants minimal returns (variance explained) with the increment of cost (overfitting). The “elbow curve” was depicted as ∑*D*_*within*_/∑*D*_*between*_, where ∑*D*_*within*_ is the sum of within cluster distances and ∑*D*_*between*_ is the sum of between cluster distances.

## (Anticipated) Results

### Demographic and Clinical Characterization

**FIGURE 2:**
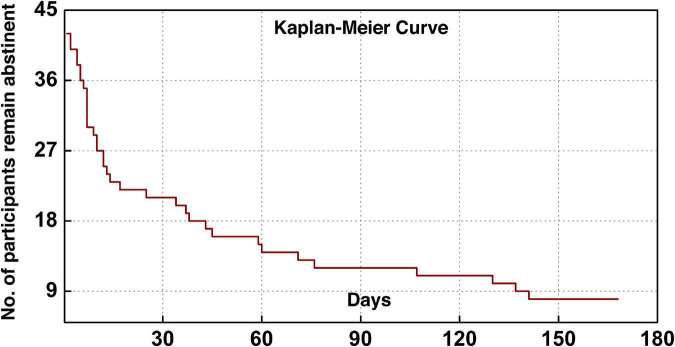
Clinical characterization of cocaine relapse. The Kaplan–Meier curve illustrates the survivorship of participants in terms of maintaining abstinence.

The current cohort of 43 participants included five females and 38 males with a mean (SD) age of 43.4 (7.2). Table 1 lists the demographic information, clinical and head-motion assessments. The clinical characterization of cocaine relapse during the follow-up is illustrated in Figure 2; as shown in the Kaplan–Meier curve, during the early relapse at cut-off of 30-day, 22 out of 43 (51.2%) failed to remain abstinent; by the end of the 6-month follow-up period, 35 out of 43 (81.4%) participants had relapsed.

### The Predicative Region-of-Interest of Cocaine Relapse

**FIGURE 3:**
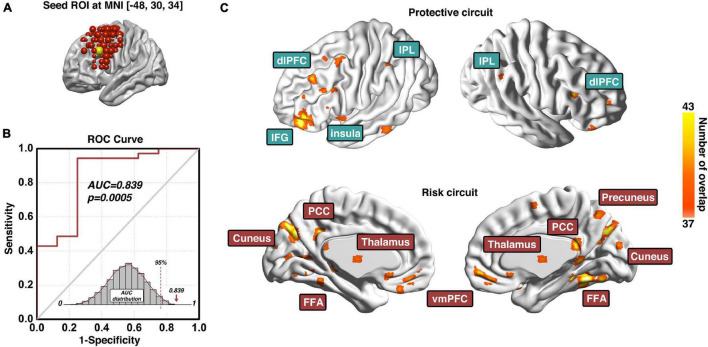
Predictive ROI location, model prediction accuracy, and associated functional circuits. Adapted from Zhai et al. (2021), panel **(A)** shows the location and MNI coordinates of the predictive dlPFC ROI; the prediction accuracy measured with the area-under-the-curve (AUC) of the receiver-operating-characteristic (ROC) curve and its corresponding statistical significance is demonstrated in panel **(B)**; and the functional circuits associated with this dlPFC ROI that were used to generate the composite indexP and indexR for subsequent early relapse prediction is recapped in panel **(C)**. ROI, region-of-interest; ROC, Receiver-Operating-Characteristics; AUC, area-under-the-curve; dlPFC, dorsolateral prefrontal cortex; IPL, inferior parietal lobule; IFG, inferior frontal gyrus; PCC, posterior cingulate cortex; FFA, fusiform face area; vmPFC, ventromedial prefrontal cortex.

The prediction modeling we proposed here will grant the final results of (1) a specific ROI (e.g., dlPFC) that is identified predictive of certain behavior (e.g., cocaine relapse), with prediction accuracy evaluated with the AUC value of the ROC curve; and (2) a set of protective circuits and risk circuits that are underlying the prediction. In our previous investigation on the dlPFC ROIs across the entire surface area of bilateral dlPFC, three dlPFC loci were identified significantly predictive of cocaine relapse with their corresponding protective and risk functional circuits (Zhai et al., 2021). Here we choose the predictive ROI on the left dlPFC to demonstrate the anticipated results of our prediction modeling pipeline (Figure 3). As Figure 3A shows, the demonstrative left dlPFC ROI is locate at MNI coordinates of [−48, 30, 34]. Figure 3B demonstrates the prediction accuracy of 83.9% as measured by the AUC value of the ROC curve. The statistical significance is confirmed by the *p*-value of 0.0005 based on the *empirical null-distribution* generated with the permutation test. The predictive ROI associated group-level protective and risk functional circuits are recapped in Figure 3C. More detailed descriptions and discussions on these functional circuits can be found in the “predictive ROI-1” section of Zhai et al. (2021). We further tested whether clinical measurements could predict cocaine relapse by utilizing the same prediction modeling method proposed here, and the clinical measurements tested here included the InDUC, CCQ, cocaine use years, days of cocaine use in the past 90 days, and days since last cocaine use. None of these measurements significantly predicted cocaine relapse (see Supplementary Table 1 for details).

### Early and Intermediate Relapse Prediction of the Predictive Region-of-Interest

**FIGURE 4:**
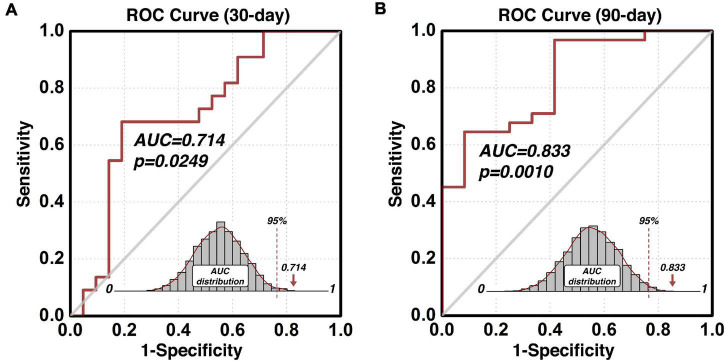
Predict validity of the early and intermediate relapse prediction models. The prediction accuracy using the functional circuits of the identified predictive dlPFC ROI is illustrated in panel **(A)** for the early relapse prediction model where the follow-up cut-off is at 30 days, and in panel **(B)** for the intermediate relapse prediction model where the follow-up cut-off is at 90 days. ROC, Receiver-Operating-Characteristics; AUC, area-under-the-curve; dlPFC, dorsolateral prefrontal cortex; ROI, region-of-interest.

The composite indices (indexP and indexR) of the protective and risk circuits associated with the predictive ROI can also be used to build other prediction models such as the early and intermediate relapse prediction models. By setting up the cut-off follow-up time at 30 days, an early relapse prediction model (Figure 4A) predicted cocaine relapse with a relatively lower, but statistically significant AUC value of 0.714 of its ROC curve. For comparison, we also built an intermediate prediction model (Figure 4B) that was capable of predicting cocaine relapse with an AUC value of 0.833 of its ROC curve by setting the cut-off follow-up time at 90 days. Statistical significance was verified with the permutation test based *empirical null-distribution* curves, which showed *p-*values of 0.0249 and 0.0010 for the early and intermediate relapse prediction models, respectively.

### *Post-hoc* Analyses of Disease Subtyping

**FIGURE 5:**
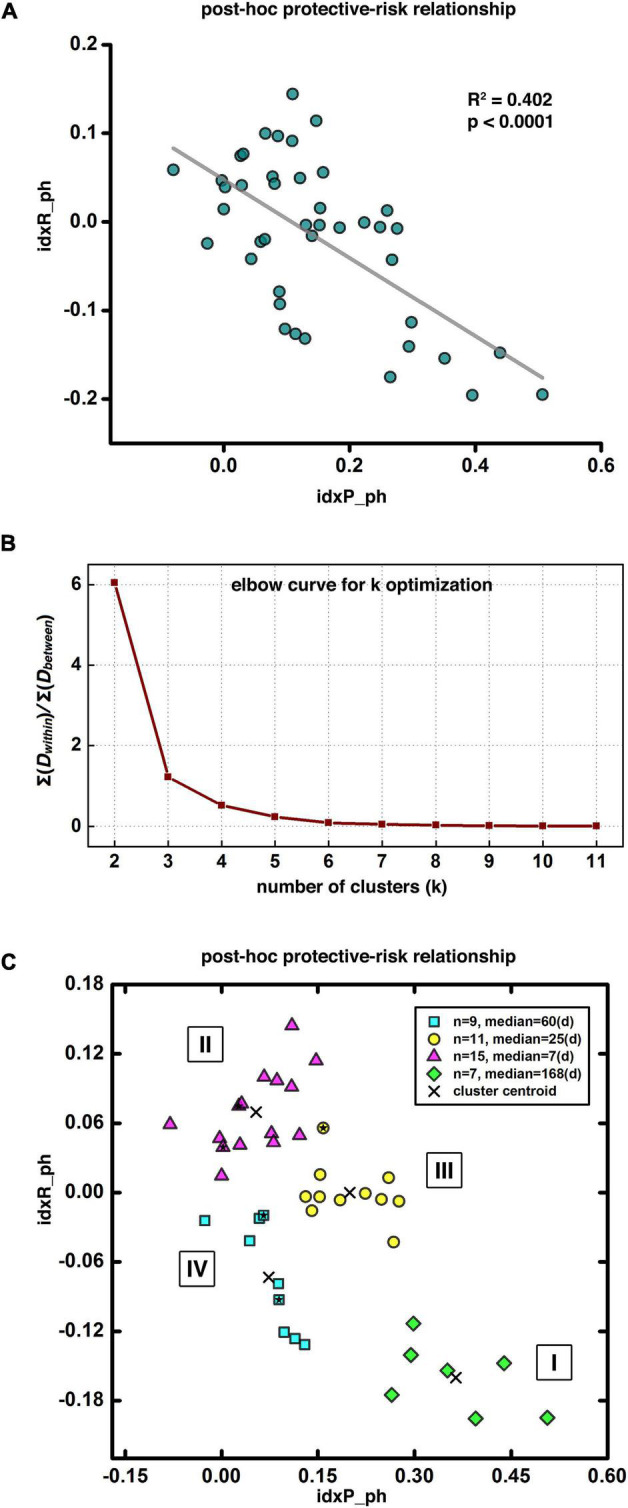
*Post-hoc* results and disease subtyping. Panel **(A)** shows significant negative correlation between the *post-hoc* protective and risk indices. Panel **(B)** demonstrates the “elbow curve” for cluster number selection in clustering analysis for disease subtyping. Panel **(C)** illustrates four potential subtypes of cocaine dependent participants as indicated by our clustering result, Subtype I at the bottom-right corner with longest abstinent days (green diamonds), Subtype II at the top-left corner with the shortest days till relapse (magenta triangles), and Subtypes III and IV in-between with moderated days till relapse (yellow circles and cyan squares). The five solid stars within the corresponding symbol shapes label the five female participants in current demonstrative cohort.

For *post-hoc* analyses exploring the protective-risk relationship, we first defined idxP_ph and idxR_ph as the averaged functional connectivity from each of the 43 participants within the group-level protective and risk circuits, respectively (note here the suffix “ph” stands for *post-hoc*, to be differentiated from the composite indices “indexP” and “indexR” used in the prediction modeling section). As Figure 5A shows, the *post-hoc* indices idxP_ph and idxR_ph showed significant negative correlation (*R*^2^ = 0.402, *p* < 0.0001). For potential subtyping of cocaine use disorder in terms of vulnerability to relapse, we also conducted the *k*-means clustering in the two-dimensional space of (idxP_ph, idxR_ph). Figure 5B shows the “elbow curve” depicted as the ratio of the sum of within cluster distances to the sum of between cluster distances from *k* = 2 to *k* = 11. Here *k* = 4 was selected as the number of clusters as it represents a good balance between the benefit and the cost based on visual inspection of the elbow curve. The clustering result is shown in Figure 5C. Each of the four clusters represents a specific subtype of cocaine dependent participants. Cluster I is at the bottom-right corner (green diamond, *n* = 7, median days till relapse = 168 days) of the (idxP_ph, idxR_ph) space, Cluster II at the top-left corner (magenta triangle, *n* = 15, median days till relapse = 7 days), and Cluster III and Cluster IV are in the middle (yellow circle, *n* = 11, median days till relapse = 25 days; and cyan square, *n* = 9, median days till relapse = 60 days). Note here all *post-hoc* analyses conducted in the (idxP_ph, idxR_ph) space were with a sample size of 42, as one participant was excluded since both *post-hoc* indices were beyond the three-standard-deviation range. Corresponding results with this participant included can be seen in Supplementary Figure 1.

## Discussion

We presented here a modeling approach to predict time-dependent follow-up behaviors by fMRI-based brain metrics, and demonstrated the utility of the approach in predicting relapse to drug use following a psychosocial treatment in a cohort of treatment-seeking cocaine users. This modeling is a combination of seed-based functional connectivity and voxel-wise Cox regression organized in a nested cross-validation fashion, which is suitable for investigation of brain-behavior relationships reliably in patient cohorts with small-to-moderate sample sizes.

### Neural Mechanisms and Individual-Level Relapse Prediction

As demonstrated in the prediction of cocaine relapse in Figure 3, our proposed modeling method is capable of identifying a prediction model in which functional connectivity of a specific brain region predicts individual’s relapse behaviors with high accuracy. The nature of the Cox regression results in two functional brain circuit sets, one protective and one risk that collectively underlie the high prediction validity. As such, these two functional circuits could be considered a system-level neural mechanism of cocaine relapse. The ROC analysis that yielded the high AUC value is within the nested cross-validation framework, which indicates the model’s prediction potential on independent participants. Although the proposed model is built upon the 6-month follow-up data, the identified protective and risk functional circuits are also capable of predicting early relapse. As shown in Figure 4, using the indices (indexP and indexR) from the same functional circuits derived from the 6-month model, prediction of early relapse (30 day) can also be achieved with a relatively lower but statistically significant prediction accuracy (AUC of 0.714).

Based on the *post-hoc* analyses, the *post-hoc* protective factor (idxP_ph) and the risk factor (idxR_ph) are negatively correlated, suggesting participants with higher protective capability tend to have lower risk factor, and *vice versa.* Further clustering analysis on the (idxP_ph, idxR_ph) space identified four subtypes of cocaine relapse related participants. Subtype I (N = 7) had highest values of the idxP_ph and lowest idxR_ph, and held the longest time till relapse (median of 168 days till relapse). Subtype II (*N* = 15), on the contrary, had lowest values of the idxP_ph and highest idxR_ph, and were all early relapse participants with the shortest time till relapse (median of 7 days till relapse). Subtypes III (*N* = 11) and IV (*N* = 9) fell between the subtypes I and II. Participants in these two subgroups showed similar levels of idxP_ph and idxR_ph with moderate days till relapse (median of 25 and 60 days till relapse, respectively). These results suggest that using the protective and risk indices, potential subtypes may be characterized for novel/independent participants by simply measuring their resting-state fMRI and calculating the values of these fMRI indices. These imaging metrics may be used to guide the design of personalized treatment strategies specific to individuals (e.g., to promote the protective circuits for participants with a high risk index but without matched protective index; or to inhibit the risk circuits for participants with a strong, intact protective index).

### Neuromodulation Implications

As in our demonstration of relapse prediction, a dlPFC ROI located at MNI coordinates [−48, 30, 34] was found to be highly predictive with its functional circuits (Figure 3). The prediction modeling proposed here is capable of identifying brain ROIs, whose functional circuits are closely related to certain behaviors/treatment outcomes. This shows the potential utility of the prediction modeling in selecting brain sites for neuromodulation-based treatment of neuropsychiatric/neurological disorders. In neuromodulation (e.g., TMS) treatment, one of the critical issues is to determine the effective stimulation site. Intuitively, in a direct search among M different brain sites with N participants in each site to evaluate treatment efficacy (a total of M × N participants), a potential optimal stimulation site would be determined by comparing the group outcomes of these M clinical studies. Furthermore, other than the stimulation location, the large parameter space (e.g., frequency, intensity, etc.) makes such clinical investigations impractically costly and time consuming to undertake in a systematic and comprehensive manner, and no such effort has been made in neuropsychiatric disorders other than medication-resistant depression (Fox et al., 2012). An alternative strategy is a two-stage approach combining neuroimaging-based search for relevant brain areas and the actual neuromodulation on these limited and specific sites. In the first stage, imaging data are collected ideally at the baseline and after a traditional treatment (non-neuromodulation, such as psychosocial treatment), and then location-specific imaging measures (e.g., functional connectivity) are identified that are related to treatment outcome. These locations are therefore considered as potential treatment sites. Then in the second stage, these candidate sites are further confirmed for their treatment efficacy with actual neuromodulation. The first stage can be done in a systematic and comprehensive manner covering a large brain area (e.g., dlPFC) while only few most relevant brain locations being tested in the second stage, thus greatly reducing the number needed for neuromodulation-based clinical investigations. Our previous study utilizing this modeling technique investigated 98 ROIs covering the entire surface of the bilateral dlPFC and identified three ROIs predictive of cocaine relapse (Zhai et al., 2021), with one on the left side being spatially proximal to an actual dlPFC stimulation site that showed promising treatment efficacy in a clinical study treating cocaine addiction (Terraneo et al., 2016). This is a perfect example of potential applications of our proposed modeling approach in the first stage for identification of potential effective TMS sites, which can then be used to guide experimental designs in the second stage for validation of these potentially effective TMS targets.

### Limitations

Several limitations should be considered regarding our analysis. We have a moderate sample size less than 50, and an unbalanced sex (five females and 38 males), which makes the dataset less ideal. However, there are practical difficulties associated with research on psychiatric diseases such as addiction, especially with a longitudinal follow-up up to 24 weeks. Combined with proper modeling and validating method, the current dataset is capable of providing at least novel hypothesis (i.e., the identified predictive ROI and its functional circuits) to be further tested clinically (i.e., neuromodulation treatment efficacy). Our sample included only five females and future work should address the possibility of gender differences.

### Conclusion

Demonstrated with a treatment-seeking cocaine addiction cohort, we presented here a prediction-modeling method that combines the hypothesis-driven seed-based functional connectivity and the Cox regression-based prediction with built-in nested cross-validation to assess treatment outcome (relapse to drug use). Other than predicting certain behaviors/treatment outcomes at individual level, specific brain regions, as well as their functional circuits, relevant to the behavioral/clinical assessments can also be identified using this modeling method. Functional connectivity of the brain circuits showing protective or risk effect on drug relapse may be used for disease subtyping. Taken together, the prediction modeling method presented here is capable of identifying brain regions and related functional circuits that are predictive of certain behavior/treatment outcome, which clearly shows neuromodulation implications and can be used for individualized prognosis and treatment protocol design.

## Data Availability Statement

The data analyzed in this study is subject to the following licenses/restrictions: Raw data associated with the current study contain personally identifiable information that could compromise the privacy of research participants if shared publicly. Codes and derived data supporting the findings of this study are available from the corresponding author contingency of institutional approval, upon reasonable request. Requests to access these datasets should be directed to YY, yihongyang@intra.nida.nih.gov.

## Ethics Statement

The studies involving human participants were reviewed and approved by Institutional Review Boards of the University of Texas Southwestern Medical Center and the Veterans Administration North Texas Health Care System. The patients/participants provided their written informed consent to participate in this study.

## Author Contributions

TZ, HG, and YY contributed to the manuscript review, revision, and conception and design of the study. YY contributed to the funding/resources acquisition and overall supervision. TZ and HG organized the database. TZ performed the statistical analyses and visualization of the results. TZ wrote the first draft of the manuscript. All authors contributed to the article and approved the submitted version.

## Conflict of Interest

The authors declare that the research was conducted in the absence of any commercial or financial relationships that could be construed as a potential conflict of interest.

## Publisher’s Note

All claims expressed in this article are solely those of the authors and do not necessarily represent those of their affiliated organizations, or those of the publisher, the editors and the reviewers. Any product that may be evaluated in this article, or claim that may be made by its manufacturer, is not guaranteed or endorsed by the publisher.

## Footnotes

1
http://afni.nimh.nih.gov/afni/

2
http://www.fil.ion.ucl.ac.uk/spm/software/spm12/
