# Supplementary material for: Cox Regression Based Modeling of Functional Connectivity and Treatment Outcome for Relapse Prediction and Disease Subtyping in Substance Use Disorder
Source: Front Neurosci. 2021 Nov 11;15:768602. doi: 10.3389/fnins.2021.768602 (PMC8632554; doi:10.3389/fnins.2021.768602)
Supplement: Supplementary file 1 [file Data_Sheet_1.pdf]

Supplementary material for

# Cox regression based modeling of functional connectivity and treatment outcome for relapse prediction and disease subtyping in substance use disorder

Tianye Zhai, PhD; Hong Gu, PhD; Yihong Yang, PhD

**Content list:**

**Supplementary Figure 1.** Post-hoc results and disease subtyping (with outlier data included)

**Supplementary Table 1.** Cocaine relapse prediction validity of the clinical measurements

**Supplementary Figure 1.** Post-hoc results and disease subtyping (with outlier data included)

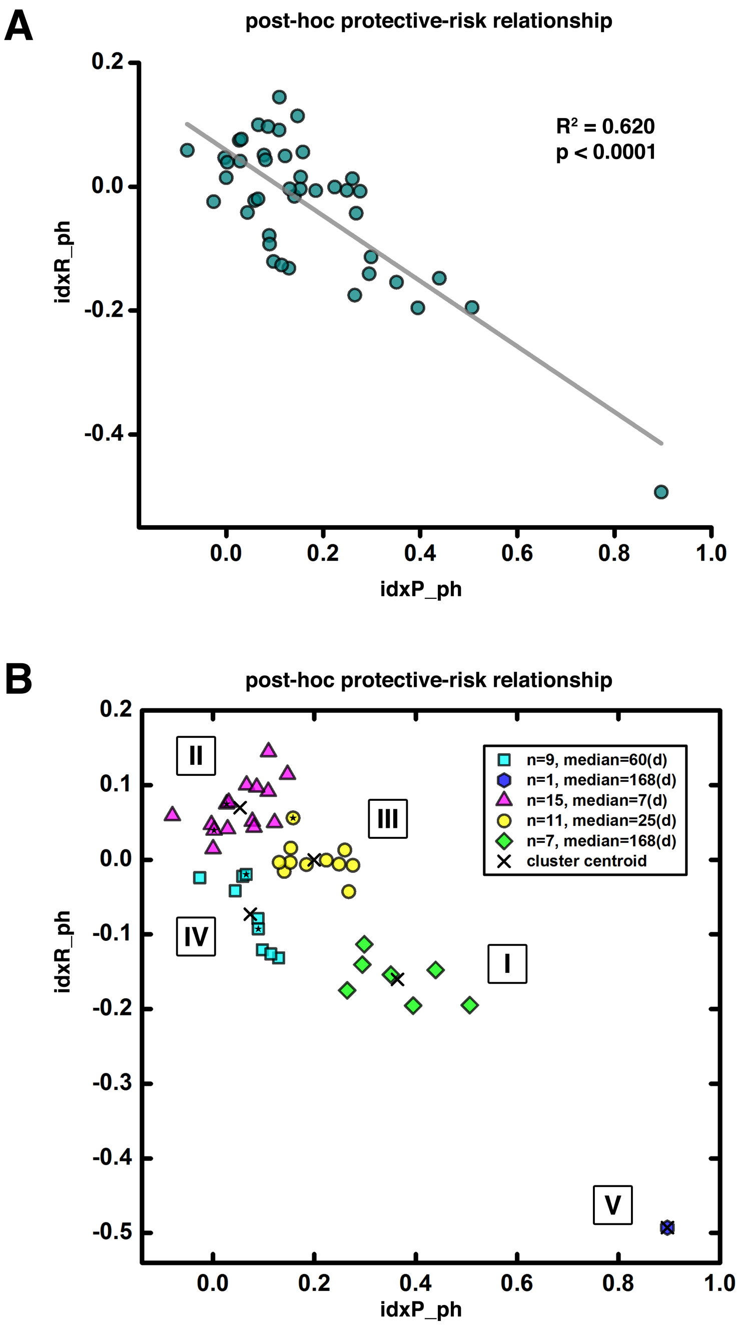


Panel **(A)** shows significant negative correlation between the post-hoc protective and risk indices. Panel **(B)** illustrates 5 potential subtypes of cocaine dependent participants as indicated by our clustering result, Subtype I (green diamonds) with long abstinent days (168 days), Subtype II at the top-left corner with the shortest days till relapse (magenta triangles), Subtypes III and IV in-between with moderate days till relapse (yellow circles and cyan squares). The outlier forms a separated Subtype V at the bottom-right corner (blue hexagon), which didn't relapse till the end of the 168-day follow-up. The five solid stars within the corresponding symbol shapes label the five female participants in current demonstrative cohort.

**Supplementary Table 1.** Cocaine relapse prediction validity of the clinical measurements

| Measurements | Index | AUC of the ROC curve | *p*-value |
|---|---|---|---|
| InDUC_90d (n=42) | Physical | 0.603 | 0.275 |
| | Interpersonal | 0.724 | 0.063 |
| | Intrapersonal | 0.570 | 0.357 |
| | Impulse Control | 0.702 | 0.094 |
| | Social Responsibility | 0.574 | 0.351 |
| InDUC_lifetime (n=42) | Physical | 0.581 | 0.321 |
| | Interpersonal | 0.522 | 0.470 |
| | Intrapersonal | 0.574 | 0.308 |
| | Impulse Control | 0.566 | 0.367 |
| | Social Responsibility | 0.540 | 0.431 |
| CCQ (n=42) | Total Score | 0.625 | 0.230 |
| Years used (n=43) | Years of cocaine use | 0.643 | 0.178 |
| Days used 90d (n=43) | Days of cocaine use in the past 90 days | 0.586 | 0.307 |
| Days since last use (n=43) | Days since last cocaine use | 0.650 | 0.171 |

*Abbreviations:* AUC, Area-Under-Curve; ROC, Receiver-Operating-Characteristics; InDUC, Inventory of Drug Use Consequences; CCQ, Cocaine Craving questionnaire
